# Changes in Parental Well‐Being Following Intensive Day Programme Treatment for Restrictive Eating Disorders in Adolescents

**DOI:** 10.1002/erv.70126

**Published:** 2026-05-23

**Authors:** Lucinda J. Gledhill, Chiara Calissano, William Thorpe, Mima Simic, Julian Baudinet

**Affiliations:** ^1^ Maudsley Centre for Child and Adolescent Eating Disorders (MCCAED) South London and Maudsley NHS Foundation Trust London UK; ^2^ King's College London, Department of Psychology Institute of Psychiatry, Psychology & Neuroscience London UK; ^3^ South West London and St George's Children and Young Person's Eating Disorder Service Springfield Hospital South West London and St George's NHS Foundation Trust London UK; ^4^ Centre for Research in Eating and Weight Disorders Institute of Psychiatry, Psychology & Neuroscience London UK

**Keywords:** adolescents, anorexia nervosa, day programme, parents, partial hospitalisation programme (PHP)

## Abstract

**Objective:**

Parents of young people with anorexia nervosa (AN) often experience significant psychological distress, including elevated levels of anxiety and depression, as well as impaired daily functioning and low self‐efficacy. This study examined changes in self‐reported levels of anxiety, depression, functional impairment, and self‐efficacy in parents of young people attending an intensive day treatment programme (ITP) for adolescents with AN.

**Method:**

Participants were 71 parents of young people enrolled in ITP. Parents completed standardised outcome measures at assessment and discharge, including the Hospital Anxiety and Depression Scale (HADS), the Work and Social Adjustment Scale (WSAS), and the General Self‐Efficacy Scale (GSE).

**Results:**

Parents reported significant reductions in anxiety, depression, and functional impairment from assessment to discharge. No significant change was found in self‐efficacy scores. No baseline factors for the young people of these parents predicted change in parental wellbeing or functioning.

**Conclusions:**

Findings suggest that ITP may contribute to modest but significant improvements in parents' mental health and daily functioning. These quantitative outcomes align with prior qualitative findings and highlight the potential value of structured, parent‐inclusive day treatment in supporting families affected by AN.

## Highlights

- Literature on parental well‐being in day programme treatment for eating disorders is limited.
- Parental anxiety, depression and functioning improved from start to end of treatment.
- No relationship was found between young person baseline factors and change in parental well‐being.

**Abbreviations**
DP: day programme
FT‐AN: family therapy for anorexia nervosa
ITP: intensive treatment programme
MCCAED: Maudsley Centre for Child & Adolescent Eating Disorders, Maudsley Hospital, UK
NICE: National Institute for Clinical Excellence

## 1 Background

Anorexia nervosa (AN) is a serious psychiatric disorder that often develops in adolescence (Bryant‐Waugh and Baudinet 2025). AN has a profound impact not only on the sufferer, but also on their family—particularly parents, who are often the primary caregivers (Treasure et al. 2001; Whitney et al. 2005). As such, in the United Kingdom, the recommended treatment for adolescent AN is Family based treatment (National Institute for Health and Care Excellence 2017). Parents are supported to use their skills to tackle the eating disorder on behalf of their child (Eisler et al. 2016; Le Grange and Eisler 2009), managing feeding, weight restoration, eating‐related behaviours, and emotion dysregulation (Austin et al. 2025; Baudinet et al. 2021a, 2021b; Eisler et al. 2016).

However, the sense of responsibility, significant emotional distress, and challenges understanding of the illness can be experienced as overwhelming for parents, resulting in a loss of normal family life, profound exhaustion, and feelings of helplessness (Denton et al. 2017; Kyriacou et al. 2008; Rhind et al. 2016). While research has indicated clinically higher rates of anxiety, depression, and overall psychological distress in this population (Forsberg et al. 2017; Kyriacou et al. 2008; Whitney et al. 2005), evidence suggests that this improves as the young person with AN recovers (Forsberg et al. 2017), and parents' sense of self‐efficacy increases (Baudinet, Eisler, et al. 2021; Baudinet et al. 2021a, 2021b; Robinson et al. 2013; Sadeh‐Sharvit et al. 2018). Greater increases in parental self‐efficacy over the initial stages of treatment have also been found to predict weight gain in family‐based treatment (Byrne et al. 2015; Sadeh‐Sharvit et al. 2018).

However, a significant proportion of young people do not respond to outpatient treatment and require inpatient admissions or intensification of outpatient treatment, such as multi‐family therapy (MFT; Baudinet, Eisler, et al. 2021; Baudinet et al. 2021a, 2021b; Baudinet and Eisler 2024), intensive outpatient programmes (IoPs), or day programmes/hospitals (DPs). Inpatient admissions are costly and associated with higher relapse rates (Meule et al. 2020; Quadflieg et al. 2023), therefore intensive outpatient treatments have increased in popularity in recent years (Baudinet and Simic 2021). These services increase the intensity of support offered to both the young person and their family system whilst allowing the young person to live at home, thereby maintaining family involvement and minimising disruption (Baudinet and Simic 2021) (Table 1).

**TABLE 1:** Baseline and discharge data for young people of parents included in this study.

		Mean (SD)	Range
Assessment	Illness duration before ITP	16.56 (10.20)	1.00–48.00
	Age	15.32 (1.58)	11.70–18.00
	%mBMI	82.37 (8.15)	66.60–101.80
Discharge	ITP treatment length (weeks)	15.78 (3.47)	5.00–24.00
	%mBMI	92.53 (7.84)	77.90–110.30

*Note:* Illness duration before ITP (months), age (years), ITP treatment length (weeks); %mBMI (percentage median body mass index).

One such day programme is the Intensive day Treatment Programme (ITP) at the Maudsley Centre for Child and Adolescent Eating Disorders (MCCAED) in London, UK. ITP treats young people aged 11–18 with restrictive eating disorders such as AN or Avoidant or Restrictive Food Intake Disorder (ARFID). Treatment focuses on weight restoration, improving eating disorder psychopathology, comorbid depression and anxiety, quality of life, interpersonal functioning, and emotional overcontrol, with positive outcomes (both quantitative: Simic et al. 2018; Baudinet et al. 2020, and qualitative: Colla et al. 2023; Gledhill et al. 2023, 2025). As part of treatment at ITP, young people attend a therapeutic group programme including CBT for anxiety and perfectionism, Radically Open Dialectical Behaviour Therapy, Compassion Focused Therapy, Distress Tolerance, an Identity Group, and Food Group (a mixture of psychoeducation around healthy eating, and practical cooking), as well as receiving weekly individual therapy aimed at improving motivation to change, and focusing on goals. Young people are supported with meals throughout the day by staff. Young people attend ITP for approximately 15 weeks, attending in a more intense capacity (5 days per week) at the start, and reducing their attendance as their symptoms begin to improve. Parental involvement includes attending weekly FT‐AN sessions as well as attending a weekly parent skills group and a weekly family meal.

While previous publications offer rich qualitative insights, it remains unclear whether these perceived changes are reflected in quantitative improvements in parents' mental health and functioning. Whilst day programmes have their benefits, it is possible that the increased intensity of these interventions may also increase burden and stress in parents/carers. In other centres, data suggest DPs can improve parental self‐efficacy (Wilson et al. 2025), perceived burden and expressed emotion (Hibbs et al. 2015; Philipp et al. 2020; Truttmann et al. 2020), as well as psychological well‐being (Wilson et al. 2025).

## 2 Aims

The present study therefore aims to explore whether the experiences described qualitatively are also evident in standardised outcome measures of anxiety, depression, general life functioning, and self‐efficacy across the course of ITP. A secondary aim was to evaluate whether certain young person baseline characteristics were associated with parental change across ITP.

## 3 Methods

### 3.1 Participants and Procedure

All parents of young people who were attending ITP between November 2020 and September 2024 completed a battery of self‐report measures as part of routine clinical care. If there were two or more parents in a family, all were invited to complete the battery. Data from families that attended ITP during the COVID pandemic have been removed from analysis as the programme offered during this time was not comparable and data have been published elsewhere (Brothwood et al. 2021).

### 3.2 Ethical Considerations

This project was approved by the South London and Maudsley Child and Adolescent Mental Health Services (CAMHS) Service Evaluation and Audit Committee.

### 3.3 Treatment

ITP operates 5 days per week (Monday to Friday) in Southeast London, UK. It is one part of the Maudsley Centre for Child & Adolescent Eating Disorders (MCCAED), a large, publicly funded, specialist child and adolescent eating disorder service. ITP is a rolling open‐ended programme with a mean length of approximately 13 weeks. Treatment involves weekly individual therapy for the young person, alongside weekly FT‐AN (Family Therapy for Anorexia Nervosa: Eisler et al. 2016) sessions focusing primarily on phase one and two of the model (development of therapeutic alliance and engagement, encouraging parents to support their young person to eat through holding boundaries with warmth, and identifying patterns that families may have become stuck in that inadvertently reinforce the illness). One to two therapy groups are attended each day, each focusing on difficulties known to co‐occur with restrictive eating disorders, such as anxiety, depression, perfectionism, emotional over‐control, behavioural/cognitive rigidity, and low self‐compassion. In addition, parents are invited to a weekly parent/skills group and a weekly family meal, as well as regular reviews with a dietician and psychiatrist if needed. See previous outcome studies for further description of the programme and treatment response (Baudinet et al. 2020; Simic et al. 2018).

### 3.4 Parent Self‐Report Measures

The General Self‐Efficacy Scale (GSE; Schwarzer et al. 1995) is a 10‐item measure assessing an individual's belief in their ability to handle difficult situations and exert control over their environment. Items (e.g., ‘I can always manage to solve difficult problems if I try hard enough’) are rated on a 4‐point Likert scale (1 = Not at all true, 4 = Exactly true), with higher scores indicating greater self‐efficacy. The GSE has demonstrated strong reliability and validity across diverse populations (Scholz et al. 2002).

The Work and Social Adjustment Scale (WSAS; Mundt et al. 2002) is a 5‐item self‐report measure assessing impairment in functioning across key life domains, including work, home management, social leisure, private leisure, and close relationships. Each item is rated on a 9‐point Likert scale (0 = Not at all impaired, 8 = Very severely impaired), with higher scores reflecting greater functional impairment.

The Hospital Anxiety and Depression Scale (HADS; Zigmond and Snaith 1983) is a 14‐item measure assessing symptoms of anxiety (7 items) and depression (7 items) in medical and psychological settings. Items (e.g., ‘I feel tense or wound up') are rated on a 4‐point scale, with separate subscale scores for anxiety and depression. Higher scores indicate greater symptom severity, with established clinical cut‐offs. The HADS has been widely validated in various clinical populations, including caregivers of individuals with eating disorders (Sepulveda et al. 2009).

### 3.5 Young Person Data

Baseline age, eating disorder diagnosis, duration of illness prior to ITP, number of comorbidities, and percentage median body mass Index (%mBMI) at assessment and discharge were extracted from case files for the included families. Additionally, length of ITP treatment was also extracted.

### 3.6 Statistical Analysis

Data were analysed using paired‐sample *t*‐tests to examine changes in parental anxiety, depression, functional impairment, and self‐efficacy from assessment to discharge. Linear regression was also used to investigate whether any patient baseline factors (age, diagnosis, comorbidities, duration of illness, %mBMI) or treatment factors (length of ITP treatment) impacted change in parental well‐being. Effect sizes were calculated using Cohen's *d*. Statistical significance was set at *p* < 0.05. Analyses were conducted using SPSS (version 31.0).

## 4 Results

### 4.1 Sample

During the recruitment period, 71 matched pairs (61 mothers; 10 fathers) completed measures at assessment and discharge. The 71 parents all came from different families (i.e., no participants were partners in the same family). No other type of caregiver (grandparent, carer, step‐parent) completed measures at both assessment and discharge.

Data were drawn from 71 young people related to each parent included in the present sample.

The majority of young people had a diagnosis (according to the Diagnostic and Statistical Manual of Mental Disorders, version 5 [American Psychiatric Association 2013]) of anorexia nervosa restrictive subtype (*n* = 63; 88.7%). Three young people had a diagnosis of anorexia nervosa binge‐purge subtype (*n* = 3; 4.2%) and four of Other Specified Feeding and Eating Disorder (OSFED) (*n* = 4; 5.6%), and one of Avoidant/Restrictive Food Intake Disorder (ARFID) (*n* = 1; 1.4%) respectively. Seventeen young people had a comorbid diagnosis (*n* = 17, 23.9%), including nine of generalised anxiety (*n* = 9; 12.7%), five of autistic spectrum condition (*n* = 5, 7.0%), three of obsessive compulsive disorder (*n* = 3, 4.2%), and one each of recurrent depressive disorder (*n* = 1, 1.4%), mixed depression and anxiety (*n* = 1, 1.4%), and misophonia (*n* = 1, 1.4%) respectively. Three young people had two comorbid diagnoses.

### 4.2 Outcomes

Cronbach's alpha were calculated for each questionnaire used. For the 5‐item WSAS and the 10‐item GSE, internal consistency was found to be good (WSAS *α* = 0.85; GSE *α* = 0.90), whereas for the 14‐item HADS, internal consistency was slightly lower although acceptable (*α* = 0.72).

Paired samples *t*‐tests revealed a significant reduction in parental WSAS scores from assessment to discharge, with a small–moderate effect size). There was also a significant reduction in parent HADS‐Anxiety scores from assessment to discharge, with a small–moderate effect size (as well as a significant reduction in parent HADS‐Depression scores from assessment to discharge, also with a small–moderate effect size. However, there was no significant improvement in GSE scores from assessment to discharge (Table 2).

**TABLE 2:** Results from paired samples *t*‐tests carried out on outcome variables at assessment and discharge.

	Assessment (*M*, SD)	Discharge (*M*, SD)	*t*‐Statistic	Significance (*p*)	Effect size (*d*)
WSAS	20.59 (8.61)	17.99 (8.43)	2.57	≤ 0.05	0.3
HADS‐Anxiety	9.66 (4.13)	8.45 (3.52)	2.45	≤ 0.05	0.3
HADS‐Depression	7.01 (4.11)	5.96 (3.25)	2.25	≤ 0.05	0.3
GSE	31.61 (4.99)	31.76 (3.94)	−0.28	0.78	—

Multiple linear regressions were carried out to investigate whether any young person baseline factors predicted change in parental WSAS, HADS‐Anxiety, HADS‐Depression, or GSE. Baseline factors of %median BMI at assessment, illness duration prior to ITP, age at assessment, and number of comorbid diagnoses were included in all regression models.

For change in GSE scores, multicollinearity was not a concern (%median BMI at assessment: Tolerance = 0.90, VIF = 1.11; Illness duration: Tolerance = 0.86, VIF = 1.17; age at assessment: Tolerance = 0.87, VIF = 1.15; comorbid diagnoses: Tolerance = 0.95, VIF = 1.05), and data met the assumption of independent errors (Durbin–Watson value = 1.83). A scatterplot of standardised residuals also showed that data met the assumption of homogeneity of variance and linearity. The linear regression model was not significant (*R* ^2^ = 0.02, *F*(6,64), = 0.18, *p* = 0.98), suggesting no baseline factors predicted change in GSE score.

For change in WSAS scores, multicollinearity was not a concern (%median BMI at assessment: Tolerance = 0.90, VIF = 1.11; Illness duration: Tolerance = 0.86, VIF = 1.17; age at assessment: Tolerance = 0.87, VIF = 1.15; comorbid diagnoses: Tolerance = 0.95, VIF = 1.05), and data met the assumption of independent errors (Durbin–Watson value = 1.64). A scatterplot of standardised residuals also showed that data met the assumption of homogeneity of variance and linearity. The linear regression model was not significant (*R* ^2^ = 0.05, *F*(6,63), = 0.55, *p* = 0.77), suggesting no baseline factors predicted change in WSAS score.

For change in HADS—Anxiety scores, multicollinearity was not a concern (%median BMI at assessment: Tolerance = 0.92, VIF = 1.09; Illness duration: Tolerance = 0.86, VIF = 1.16; age at assessment: Tolerance = 0.91, VIF = 1.10; comorbid diagnoses: Tolerance = 0.96, VIF = 1.04), and data met the assumption of independent errors (Durbin–Watson value = 1.19). A scatterplot of standardised residuals also showed that data met the assumption of homogeneity of variance and linearity. The linear regression model was not significant (*R* ^2^ = 0.11, *F*(6,60), = 1.18, *p* = 0.33), suggesting no baseline factors predicted change in HADS‐Anxiety score.

For change in HADS—Depression scores, multicollinearity was not a concern (%median BMI at assessment: Tolerance = 0.92, VIF = 1.08; Illness duration: Tolerance = 0.86, VIF = 1.16; age at assessment: Tolerance = 0.91, VIF = 1.10; comorbid diagnoses: Tolerance = 0.96, VIF = 1.04), and data met the assumption of independent errors (Durbin–Watson value = 1.86). A scatterplot of standardised residuals also showed that data met the assumption of homogeneity of variance and linearity. The linear regression model was not significant (*R* ^2^ = 0.09, *F*(6,60), = 1.01, *p* = 0.43), suggesting no baseline factors predicted change in HADS‐Depression score.

## 5 Discussion

This study explored changes in parental anxiety, depression, functional impairment, and self‐efficacy over the course of ITP for adolescents with anorexia nervosa. Findings showed statistically significant reductions in self‐reported anxiety, depression, and functional impairment among parents from assessment to discharge, but no significant change in general self‐efficacy. These findings align with previous research highlighting the psychological toll of caregiving in eating disorders (Forsberg et al. 2017; Kyriacou et al. 2008; Whitney et al. 2005), while offering evidence that treatment participation may contribute to improvements in parents' mental health. As shown in qualitative accounts, parents described ITP as an emotionally intensive but ultimately supportive space (Gledhill et al. 2025). Many reflected on the helpful value of the structure, boundaries and clear expectations of the programme alongside a strong therapeutic alliance with staff which supported their understanding of the illness and learning skills to manage meals and difficult emotions. It is our hypothesis that these are all potential mechanisms underlying the observed improvements in emotional outcomes, however these mechanisms would need to be investigated further in future controlled studies. The presented data can only show an association between ITP and improvements in parental well‐being/functioning, and authors are acutely aware that we cannot infer causality between ITP and these improvements.

In contrast to parental well‐being, self‐efficacy did not significantly change in the current study. This is in direct contrast to previous literature that suggests that over the course of treatment, parental self‐efficacy increases (Baudinet, Eisler, et al. 2021; Baudinet et al. 2021a, 2021b; Byrne et al. 2015; Eisler et al. 2000; Girz et al. 2013; Le Grange and Eisler 2009; Rienecke and Richmond 2018; Robinson et al. 2013; Sadeh‐Sharvit et al. 2018; Wilson et al. 2025). However, pre‐ITP scores suggest that parental self‐efficacy was already relatively high (*M* = 31, with a maximum score of 40), with parents believing that they could already manage their young person's difficulties. As there is no mid‐treatment score available it is not clear whether self‐efficacy remained constant through the intervention, or if it reduced and improved again. ITP offers clinician delivered meal support to young people throughout the day (approximately 6 hours per day), with parents being responsible for feeding their child breakfast, dinner, and all meals over the weekend. Some studies have raised concerns that clinicians assuming responsibility for feeding in PHP or IOP settings may undermine parental self‐efficacy (Hoste 2015), and that parents can experience significant difficulty when asked to adopt refeeding practices that feel unfamiliar or inconsistent with their usual parenting approach (Waage et al. 2025). However, consistent with previous findings (Van Huysse et al. 2022), the present data suggest that providing this level of clinical support does not in itself diminish parental self‐efficacy. Indeed, it would be valuable for future research to examine whether parental self‐efficacy increases further following discharge from ITP, as parents transition into later phases of treatment—typically phases 3 and 4 of the FT‐AN model—where responsibility is progressively returned to the young person and family (Eisler et al. 2016).

This study also adds to the limited literature on parent outcomes in adolescent eating disorder day programmes. Baudinet, Eisler, et al. (2021) and Baudinet et al. (2021a, 2021b) noted that although day treatment models are increasingly used, there is limited reporting on the impact on parents within these settings. Our findings complement previous studies on ITP (Baudinet et al. 2020; Colla et al. 2023; Gledhill et al. 2023, 2025; Simic et al. 2018, 2022; Stewart et al. 2022), which demonstrated promising patient outcomes, by showing that there is an association between the ITP intervention and parents experiencing meaningful psychological changes over the course of treatment (however again, causality cannot be inferred based on the data presented). This is particularly relevant given the centrality of parental involvement in eating disorder focused family therapy (Eisler et al. 2000; Baudinet, Eisler, et al. 2021; Baudinet et al. 2021a, 2021b; Blessitt et al. 2020) and the emphasis on collaborative, empowering, formulation‐driven approaches in the Maudsley FT‐AN Model (Eisler et al. 2016; Baudinet, Eisler, et al. 2021; Baudinet et al. 2021a, 2021b).

Several limitations should be acknowledged. First, the lack of demographic information limits the ability to understand how parent characteristics (e.g., socioeconomic background, ethnicity) may have influenced the outcomes. In addition, the sample is made up of 86% mothers which makes generalisability of findings challenging. It is not clear at present whether the change in well‐being is at all related to gender, or whether it is influenced by other factors. Data on the involvement in treatment of the parent completing the self‐report measures were also not available, that is, were the parents completing the measures those that were heavily involved in treatment, or the contrary, and it is unclear whether if there were a second parent in the home whether this parent would have also (a) experienced the same level of difficulty with well‐being/functioning at assessment, and (b) experienced the same level of improvement in wellbeing/functioning. A third limitation is the measures used may not have been sensitive to illness‐specific experiences—particularly the General Self‐Efficacy Scale, which may have lacked relevance to the parent context. Previous studies using the Patient versus Anorexia scale (PVA: Rhodes et al. 2005) have found significant improvements over the course of treatment, indicating this may be a preferable measure to use in future research. A fourth limitation is the lack of extra baseline characteristics data of the young people of included parents, that is, symptom severity or medical risk. It would have been interesting to see if parents of young people with more severe presentations had greater mental health/functioning difficulties at baseline, and how change in these measures differed depending on these extra baseline characteristics.

### 5.1 Conclusion

The data presented in this study show that whilst we can't infer causality, there is an association between DP treatment for adolescent restrictive eating disorders and improvements in the psychological wellbeing of parents, including improvements in symptoms of anxiety, depression, and general functioning. Self‐efficacy was unchanged, however remained high throughout treatment. No young person baseline factors predicted improvements in well‐being, functioning or self‐efficacy. This study provides some evidence for an association between DP treatment and improvements in parental well‐being, as well as providing some potential support to the view that intensifying treatment such as PHP/DP/IOP does not reduce self‐efficacy of parents.

## Funding

The authors have nothing to report.

## Ethics Statement

This study was approved by the South London and Maudsley Child and Adolescent Audit and Service Evaluation Committee. All methods were performed in accordance with the required guidelines and regulations.

## Consent

Service evaluation approval allows for analysis and publication of anonymised data generated by approved projects.

## Conflicts of Interest

The authors declare no conflicts of interest.

## Data Availability

The data that support the findings of this study are available on request from the corresponding author. The data are not publicly available due to privacy or ethical restrictions.
